# Supplementary material for: Functional Characterization of Three Diterpene Synthases Responsible for Tetracyclic Diterpene Biosynthesis in Scoparia dulcis
Source: Plants (Basel). 2022 Dec 23;12(1):69. doi: 10.3390/plants12010069 (PMC9824296; doi:10.3390/plants12010069)

Supplementary material
for

Functional Characterization of Three Diterpene Synthases Responsible for Tetracyclic Diterpene Biosynthesis in *Scoparia dulcis*

Jung-Bum Lee*, Tomoya Ohmura, and Yoshimi Yamamura

Graduate School of Medicine and Pharmaceutical Sciences,
University of Toyama, 2630 Sugitani, Toyama, Toyama,
930-0194, Japan

Content:

Methods
Figure S1. Amino acid alignments of DTSs from *Scoparia dulcis*.
Figure S2. ^{1}H NMR spectrum of *syn*-copalol (**3'**) in $CDCl_3$
Figure S3. ^{13}C NMR spectrum of *syn*-copalol (**3'**) in $CDCl_3$
Figure S4. HMQC spectrum of *syn*-copalol (**3'**) in $CDCl_3$
Figure S5. HMBC spectrum of *syn*-copalol (**3'**) in $CDCl_3$
Figure S6. ^{1}H NMR spectrum of scopadula-13α-ol (**4**) in $CDCl_3$
Figure S7. ^{13}C NMR spectrum of scopadula-13α-ol (**4**) in $CDCl_3$
Figure S8. COSY spectrum of scopadula-13α-ol (**4**) in $CDCl_3$
Figure S9. HMQC spectrum of scopadula-13α-ol (**4**) in $CDCl_3$
Figure S10. HMBC spectrum of scopadula-13α-ol (**4**) in $CDCl_3$
Figure S11. ^{1}H NMR spectrum of aphidicol-16-ene (**8**) in $CDCl_3$
Figure S12. ^{13}C NMR spectrum of aphidicol-16-ene (**8**) in $CDCl_3$
Figure S13. COSY spectrum of aphidicol-16-ene (**8**) in $CDCl_3$
Figure S14. HMQC spectrum of aphidicol-16-ene (**8**) in $CDCl_3$
Figure S15. HMBC spectrum of aphidicol-16-ene (**8**) in $CDCl_3$
Figure S16. NOESY spectrum of aphidicol-16-ene (**8**) in $CDCl_3$
Table S1. Primer sequences used in the present study
Table S2. Abbreviations and accession numbers of DTSs
Table S3. ^{1}H and ^{13}C NMR assignments of *syn*-copalol (**3'**) in $CDCl_3$
Table S4. ^{1}H and ^{13}C NMR assignments of scopadula-13α-ol (**4**) in $CDCl_3$
Table S5. ^{1}H and ^{13}C NMR assignments of aphidicol-16-ene (**8**) in $CDCl_3$

Methods

The final dephosphorylated SdCPS2 product **3'**, SdKSL1 product **4**, and SdKSL2 product **8** were obtained from large-scale culture after purification on silica gel column chromatography with hexane-acetone gradient system. The final yields of pure **3'**, **4**, and **8** were estimated to be ~3 mg. Those compounds were dissolved in 0.5 mL of $CDCl_3$ (Aldrich). NMR spectra were acquired on a JEOL ECA-500 spectrophotometer. All NMR experiments were carried out at 25 ˚C. Chemical shifts were calculated by reference to those known for $CDCl_3$ signals offset from TMS (^{13}C 77.23 ppm, ^{1}H 7.24 ppm). All spectra were acquired using standard programs from the Delta software, with collection of 1D ^{1}H-NMR, and 2D heteronuclear single-quantum coherence (HSQC), heteronuclear multiple-bond correlation (HMBC), heteronuclear 2-bond correlation (H2BC), as well as 1D ^{13}C-NMR, spectra.

Additional Figures

```
SdCPS1      1 MALATFLRSPPSPGVFAAALHVPSSS---TFGRTTTYVFPSYSWKTPV  45
SdCPS2      1 MG-SSHLSATHRPG----GVRLPSEQLCRIWQKGRLY-----------  32
SdKSL1      1 MS----------------------------------------------   2
SdKSL2      1 L-----------------------------------------------   1
SdKSL2mut   1 L-----------------------------------------------   1

SdCPS1     46 TSSNTSPCNA------ISRPRTEERIDLIRNGFPTINYWHEIVEDDTQ  87
SdCPS2     33 EATSKLPCSTYHN-IWISKPNGD----SSRIGDVSIRS-QVYVQDYQH  74
SdKSL1      3 CQTKIIPISNYQTGIWTSKHYSS-------------------VDSFPR  31
SdKSL2      2 ----FVYLWKYQTGIWTGKHYSS-------------------VDALPR  26
SdKSL2mut   2 ----FVYLWKYQTGIWTGKHYSS-------------------VDALPR  26

                                                  SxYDTAW
SdCPS1     88 LEITHRETAISSNKIWEMVKTIRGMLRSMEDGEITVSAYDTAWVALVE 135
SdCPS2     75 LEQEAME-EISEIKMQDCISSIKSSLNSIDEGQISVSAYDTAWVALIR 121
SdKSL1     32 KGRSIIV-KCNSQSIQDSTEKIREMLSTNAKIEISPSAYDTAWVAMVP  78
SdKSL2     27 KDRLIIV-KCNSQSIQDSTEKIKEMLSTNAKIEISPSAYDTAWVAMVP  73
SdKSL2mut  27 KDRLIIV-KCNSQSIQDSTEKIKEMLSTNAKIEISPSAYDTAWVAMVP  73

                                   QxxDGSW
SdCPS1    136 DIGG--SGTPQFPSSLEWISNNQLPDGSWG--DGATFSAHDRIINTLA 179
SdCPS2    122 DLEG--GDCPQFPSSLEWIANNQLSDGSWG--YEDFFLVYDRLVCTLA 165
SdKSL1     79 SSPGYSGGKPFFPQCLDWILENQNPDGSWGLDPGHPSLVKDSLSCTLA 126
SdKSL2     74 SSPRYSGGKPFFPQCLDWIMENQNPDGSWGLDPGHPSLVKDSLSSTLA 121
SdKSL2mut  74 SSPRYSGGKPFFPQCLDWIMENQNPDGSWGLDPGHPSLVKDSLSSTLA 121

SdCPS1    180 CVIALRTWNMHSDKSDRGIAFIRENMYKLEDENEEHMPIGFEVALPSL 227
SdCPS2    166 CVIALKSWNIHIDKIERGILFIKENMNTLEDANPENMTCGFETVFPVL 213
SdKSL1    127 CLLALRKWNLGQQQIHKGLDYIGTNGCAICDKDQVS-PIGFDIIFPSM 173
SdKSL2    122 CLLALRKWNFGQQQIQKGLDYIGSNEWAICDKDQVS-PIGFDIVFPSM 168
SdKSL2mut 122 CLLALRKWNFGQQQIQKGLDYIGSNEWAICDKDQVS-PIGFDIVFPSM 168

SdCPS1    228 IEIAKKIDI---D-IPDESPIFEKRLRKKRFKLKRIPRDIMHKVPTTL 271
SdCPS2    214 LHKAKDLGI---EGIPYDASVIQHISAERDRKIQRVPKELMHEIATCM 258
SdKSL1    174 VNSAREMGLVMPDSIKLDTSIYNMLDP---RYVSLLGNDCIASKNQIL 218
SdKSL2    169 VNSARKMGLVLADPIILDTSIYNIMDP---RYASLLGNDHFPSKNQIL 213
SdKSL2mut 169 VNSARKMGLVLADPIILDTSIYNIMDP---RYASLLGNDHFPSKNQIL 213

SdCPS1    272 LHSLEGMP--ALDWEKLINLQS-ADGSFLFSPSSTAFALQQTKDHNCL 316
SdCPS2    259 LFNLEGLEDLGLDWQKLLKLTA-PKGSFLTSPASTAFAIINTKNEDCV 305
SdKSL1    219 GYVAEGLGKYSCNWNELLSTQQRSNGSLFNSPATTAAALIHRHDGKCL 266
SdKSL2    214 GYVAEGLEKYSCN*NELLSTQQRSNGSLFNSPATTAAALIHRHDGKCL 260
SdKSL2mut 214 GYVAEGLEKYSCNWNELLSTQQRSNGSLFNSPATTAAALIHRHDGKCL 261

SdCPS1    317 HYPARHLQKFNGGVPNVYPVDLFEHLWAVDRLERLGLSRYFQPEIEEC 364
SdCPS2    306 AYIQNIVDKCNGGAPPNYPVDIYDRLWAVDRIERLGISRFFVSEIRAC 353
SdKSL1    267 EYLLSIQKIHKTWVPTIHPMDIYARLCMIDTLERLGIRRHFEQEIGSI 314
SdKSL2    261 EYLQSILKIHKTWVPTINPMNIYARLCMIDTLERLGICRHFEQEIGSI 308
SdKSL2mut 262 EYLQSILKIHKTWVPTINPMNIYARLCMIDTLERLGICRHFEQEIGSI 309

                                     DxDD
SdCPS1    365 IDYVHGHWTSKGICWARNSE-VKDIDDTAMGFRLLKLHGYEVSADVFK 411
SdCPS2    354 LNHIYRYWSDKGLYCAGDSE-FVDIDDTSMSVRLLRLHGYNITPNALN 400
SdKSL1    315 LDQTYRYWQQE------DEEIFSDVTCLALAFRLLRMHGYEISPDKLV 356
SdKSL2    309 LHQTYRYWQQE------DEEIFSDVTCLALAFRLLRMQGYEVSSDELA 350
SdKSL2mut 310 LHQTYRYWQQE------DEEIFSDVTCLALAFRLLRMQGYEVSSDELA 351

SdCPS1    412 HFENGGEFFCFVGQSTQAVTGMYNLFRASQVMFPGED-ILSNAKSFLS 458
SdCPS2    401 NFKKDNAFTCYVGQQFESPSPLFNLYRTSQILYPGET-ILEEAKEFTY 447
SdKSL1    357 AFSEEESFFNTTSIQFTGIPTILELYRASEVALDEEEIILDKIQAWTS 404
SdKSL2    351 AFSEEESFFNTTMIQFTGIHTILELYRASEVALDEEEIVLDKIQAWTS 398
SdKSL2mut 352 AFSEEESFFNTTMIQFTGIHTILELYRASEVALDEEEIVLDKIQAWTS 399

SdCPS1    459 KFLQEKRANNELLDKWIITKDLPGEVGYALDVPWYASLPRVETRLYLE 506
SdCPS2    448 NFLKERLESNQVLDKWLISKKLPDEIRHGLEMPWYASLPRLETRFYLE 495
SdKSL1    405 KYLKQK-----LLDHSISDKRLHKQVEYAMET-FYGTLDRVEHRRTIE 446
SdKSL2    399 KYLKQK-----LLDHSIPDKTLHKQVEYAMET-LYGTLHRVEHRRTIE 440
SdKSL2mut 400 KYLKQK-----LLDHSIPDKTLHKQVEYAMET-LYGTLHRVEHRRTIE 441
```

Figure S1: Amino acid alignments of DTSs from *S. dulcis*.

```
SdCPS1     507 QYGGQDDVWIGKTLYRMPYVNNNTYLELAKLDYNNCQASHQQEWKSIQ 554
SdCPS2     496 DYCA-DDVWIGKALYSMPNINNKAYLDLAKLDYNRCQEQHQREWNLMQ 542
SdKSL1     447 LYNT-NNFRMSKTAYRCPMIENPSFLSLAHIDFLMDRAQQQKELKQLQ 493
SdKSL2     441 LYNT-INFRMSKTAYRCPTIENPSFLSLAHNDFLMDRAQQQKELKQLQ 487
SdKSL2mut  442 LYNT-INFRMSKTAYRCPTIENPSFLSLAHNDFLMDRAQQQKELKQLQ 488

SdCPS1     555 KWYSRCNFGEYGMSERSLLLAYYIAAASIFEPERALERLAWAKTAILM 602
SdCPS2     543 QWYEKSYLQEFGITEKDLILAYFLASASIFEPERLGERVAWVKSQIVL 590
SdKSL1     494 RWCTDSRLEELKRGRNILLLSHYLSSAILVGPELSDARISYSQGIVLT 541
SdKSL2     488 RWCTDSRFKELKRGRHVLLLSHFLASAILVGPELSDARISYSQAIVLT 535
SdKSL2mut  489 RWCTDSRFKELKRGRHVLLLSHFLASAILVGPELSDARISYSQAIVLT 536

SdCPS1     603 ETIETILKDNSSQES---------------GNVLSSMNPNLMAAACMQ 635
SdCPS2     591 Q----ILLDYCSPSD---------------QNGKGNKNVEKITAFLLE 619
SdKSL1     542 TFLDDFFDKYASLKELLEFVEFINKWDGAPTTGYRTKELEIHFQAIYN 589
SdKSL2     536 TFIDDFFDKYASMKELLEFVEFINKWDRVPTTDYHTKELEIHFQAVYN 583
SdKSL2mut  537 TFIDDFFDKYASMKELLEFVEFINKWDRVPTTDYHTKELEIHFQAVYN 584
                  DDxxD

SdCPS1     636 TEQVAGITKQERLTETLVRASKPLLIGTRCWHTAGT----SISNCIMG 679
SdCPS2     620 TLVQLKMDAQEQIGRD---------IGDLLYDAWGV------------ 646
SdKSL1     590 NANELAYKASIRQGRN---------VKDYLVGIWLVCAKSQLKQVDWT 628
SdKSL2     584 NVNELASKASIRQGRN---------VKDYLVGIWLDYVNGNLKQVDWT 622
SdKSL2mut  585 NVNELASKASIRQGRN---------VKDYLVGIWLDYVNGNLKQVDWT 623

SdCPS1     680 RKSGLRTCEERDVHSGQGDAELLVRTLNLCGGGGRWVSEELLLSHPKY 727
SdCPS2     647 ---WLKRLGENEEES-QESIEPIVGTINIC--GGHIASKEFLFSHPQC 688
SdKSL1     629 TNNIIPTMEEQLLNASETIACNVVILITLLF-LGEKLSEELLHSE-EC 674
SdKSL2     623 ANNIIPTMEELLLNATMTIACNIVILIPNLF-LGEKLSEELLHSE-EC 668
SdKSL2mut  624 ANNIIPTMEELLLNATMTIACNIVILIPNLF-LGEKLSEELLHSE-EC 669

SdCPS1     728 QHLLEVTN---RVCHQLRLFQR----------------RQACDVNGGC 756
SdCPS2     689 TALSVLTS---KICHQLREVSE----------------NSNGKVLGFE 717
SdKSL1     675 GSLLYLASLLVRLLNDLQTFKREREGSERIINRVNFLLDRGGGAISEE 722
SdKSL2     669 NSLLYLASLHVRLLNDLQTFKREREDSERNINCVDFLVHRGEGAINEE 716
SdKSL2mut  670 NSLLYLASLHVRLLNDLQTFKREREDSERNINCVDFLVHRGEGAINEE 717
                                NSE/DTE

SdCPS1     757 MTDVGGILSAQIESNMQELAKLVPTKSSSGDLDSDTKQNFLTVARSFY 804
SdCPS2     718 SKD--SIIGREIENNMQSLVQLV-FQQEPGAISKDIKQIFFGVARTFY 762
SdKSL1     723 EAV--ATVKEMIKTHTRKLLKMV-VQTKGSGLSRECKNLFWNSVRIAY 767
SdKSL2     717 EAV--TTIKEMIKTYTRKLLKMV-VQTEGSKLPRECKNLFWNSVRIAY 761
SdKSL2mut  718 EAV--TTIKEMIKTYTRKLLKMV-VQTEGSKLPRECKNLFWNSVRIAY 762

SdCPS1     805 YAVYCNPG------SINFHIAKVLFERV--------L            827
SdCPS2     763 YKAYFSAE------QIDFHLSKVLFEKV--------V            785
SdKSL1     768 YLYRQSDELTNTQAKTKLDMEAVMYEPLNLSSHKLAS            804
SdKSL2     762 YLYRQSDELTNTQAKTKRDMEVVMYEPLNLSSHKLAT            798
SdKSL2mut  763 YLYRQSDELTNTQAKTKRDMEVVMYEPLNLSSHKLAT            799
```

Figure S1: Amino acid alignments of DTSs from *S. dulcis*. (*continued*)

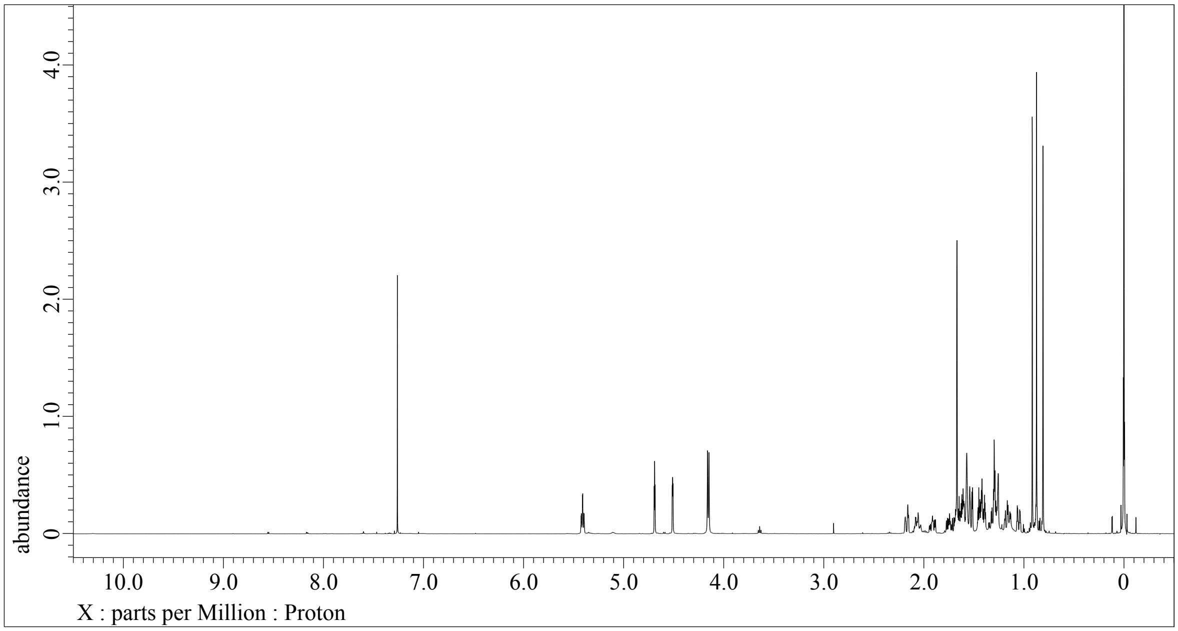

Figure S2: ^{1}H NMR spectrum of *syn*-copalol (**3'**) in $CDCl_3$

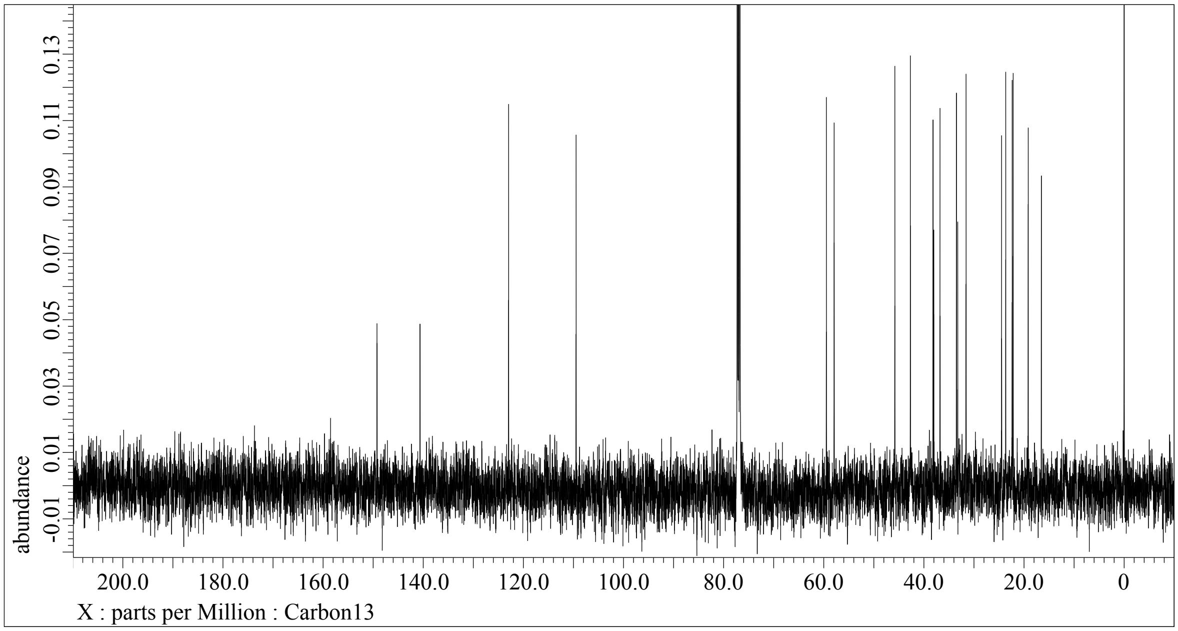

Figure S3: ^{13}C NMR spectrum of *syn*-copalol (**3'**) in $CDCl_3$

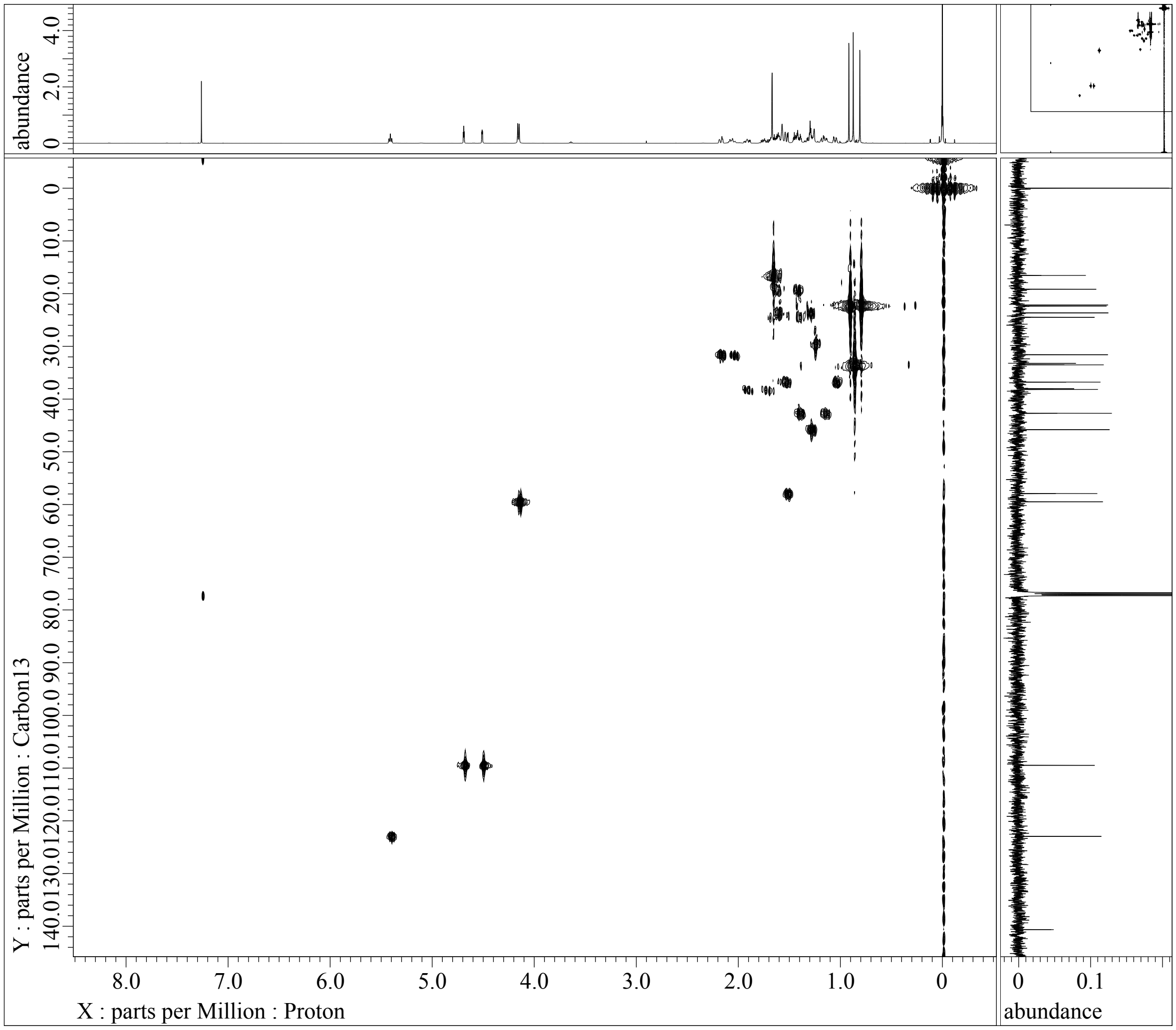

Figure S4: HMQC spectrum of *syn*-copalol (**3'**) in $CDCl_3$

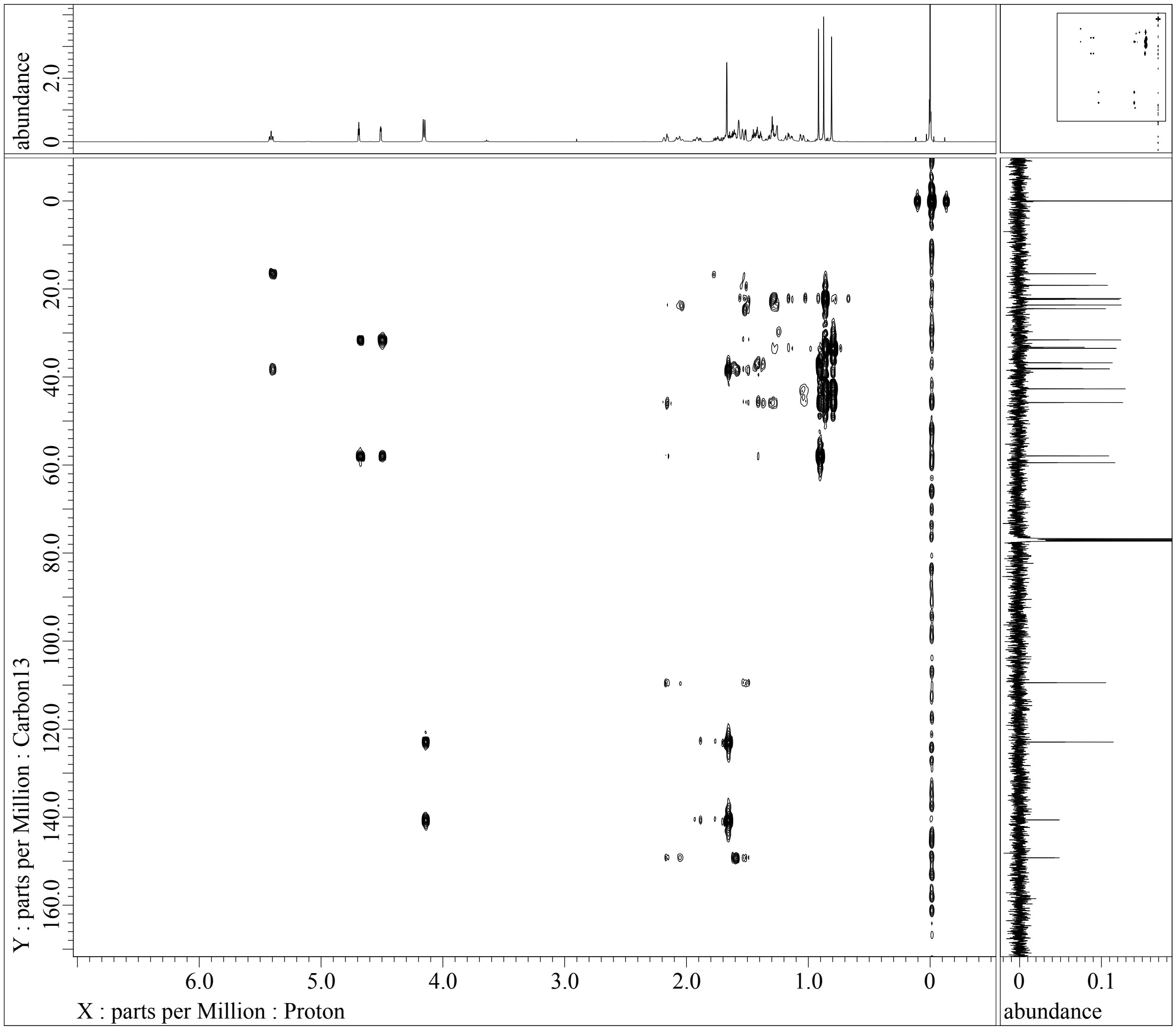

Figure S5: HMBC spectrum of *syn*-copalol (**3'**) in $CDCl_3$

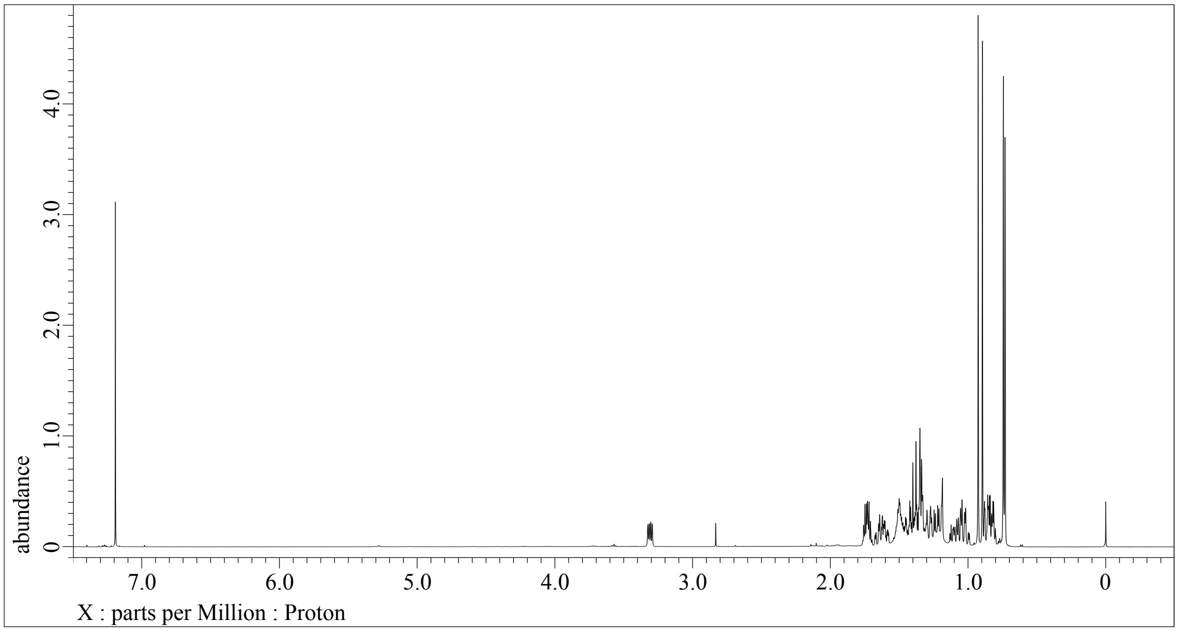

Figure S6: ^{1}H NMR spectrum of scopadula-13α-ol (**4**) in $CDCl_3$

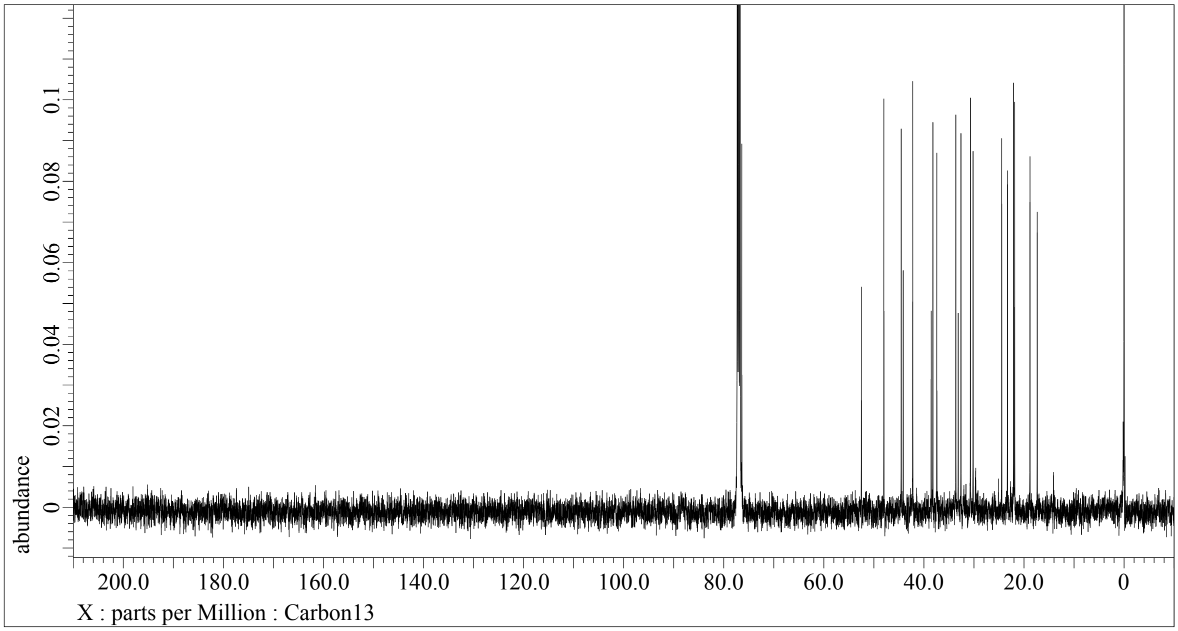

Figure S7: ^{13}C NMR spectrum of scopadula-13α-ol (**4**) in $CDCl_3$

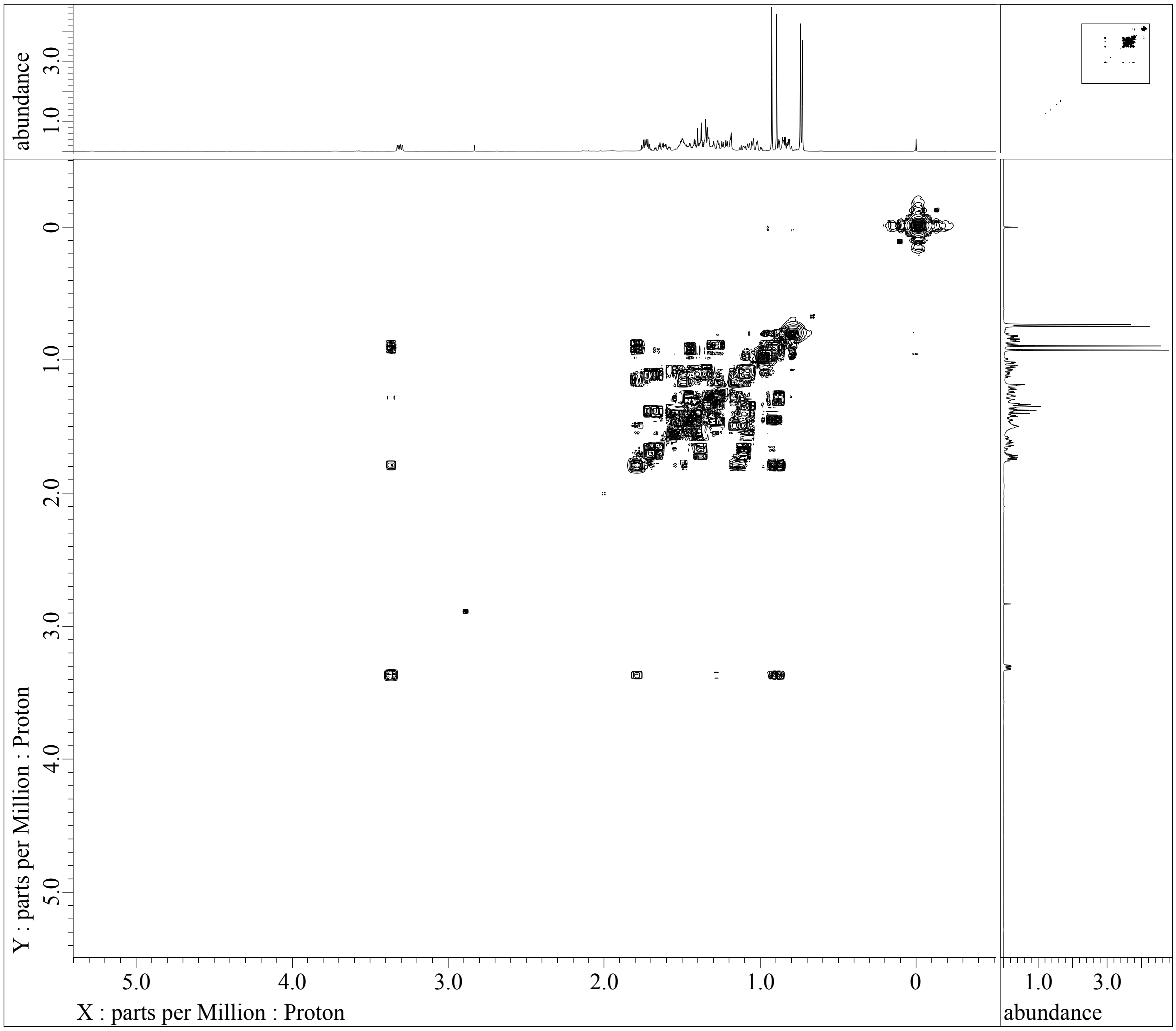

Figure S8: COSY spectrum of scopadula-13α-ol (**4**) in $CDCl_3$

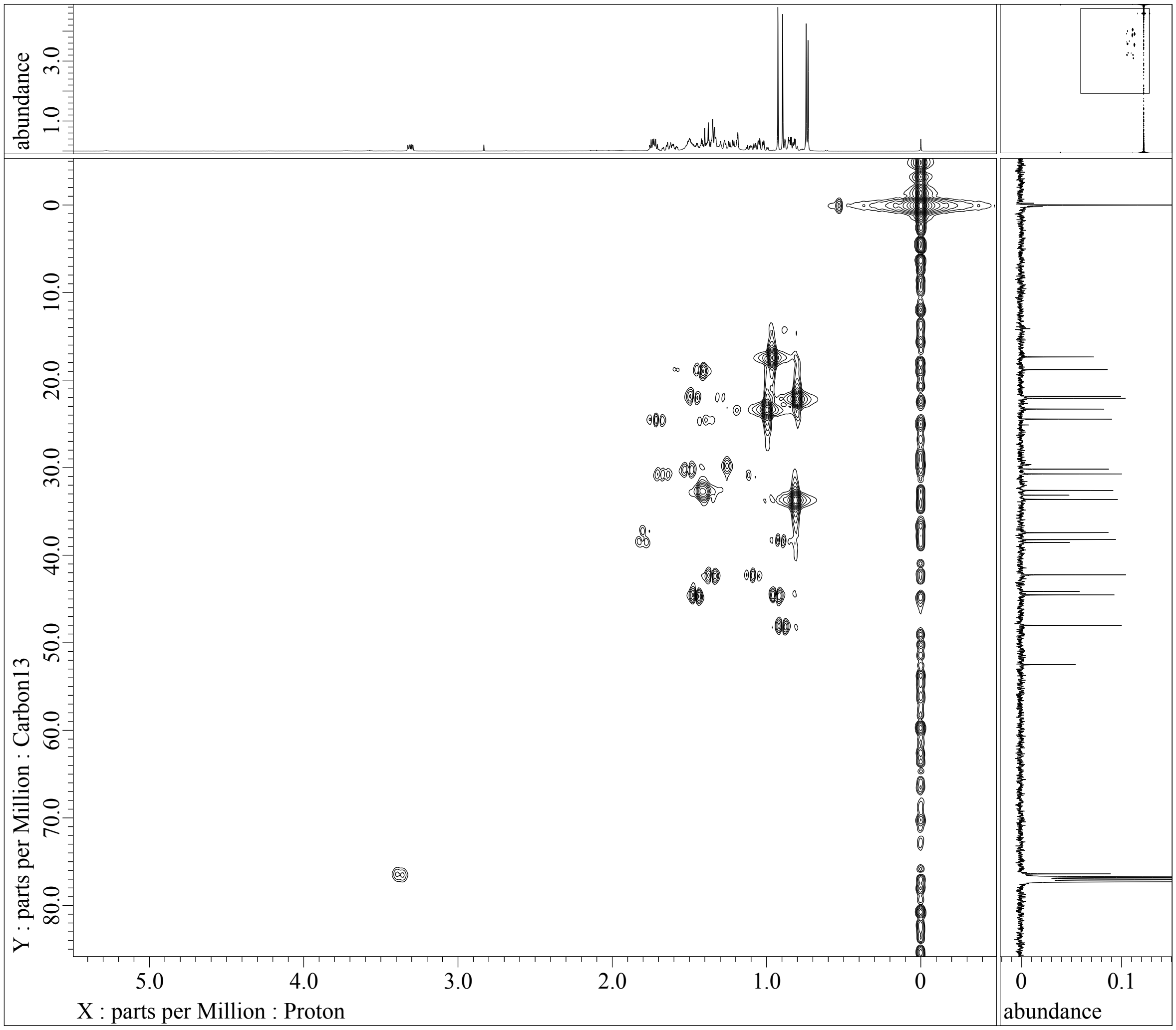

Figure S9: HMQC spectrum of scopadula-13α-ol (**4**) in $CDCl_3$

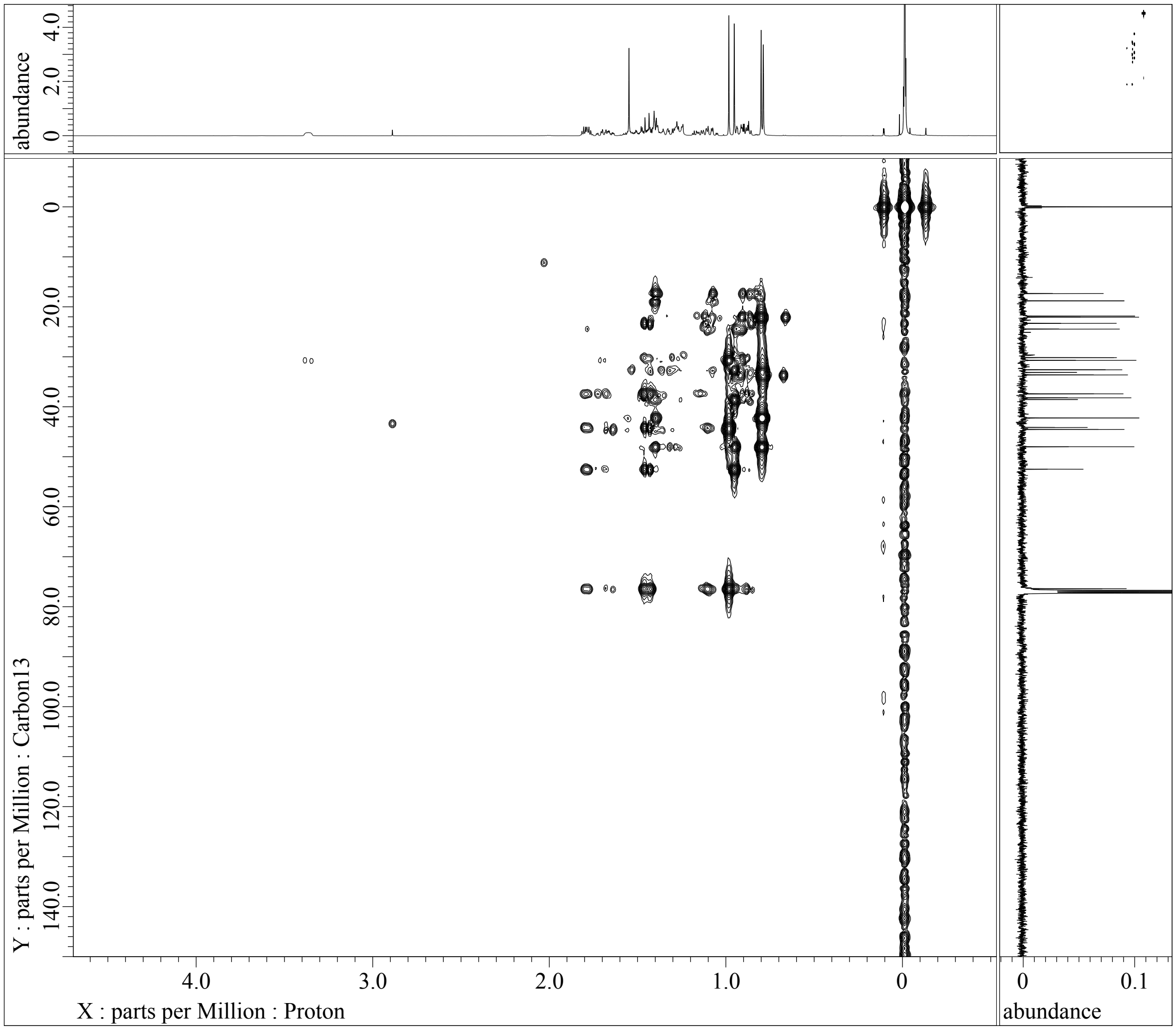

Figure S10: HMBC spectrum of scopadula-13α-ol (**4**) in $CDCl_3$

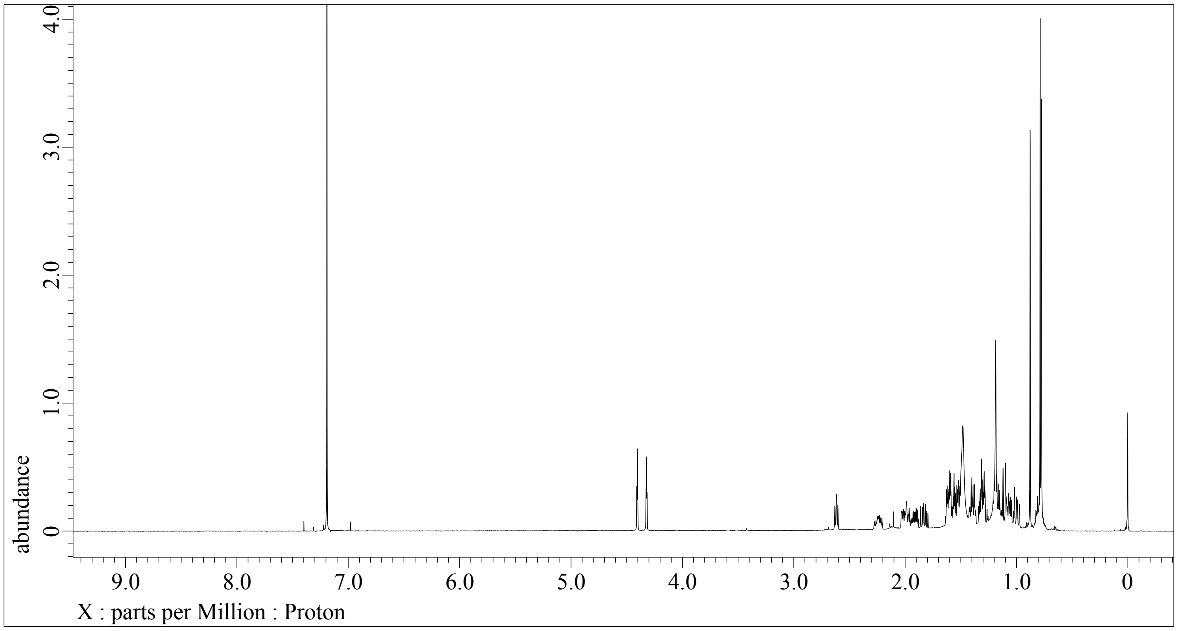

Figure S11: ^{1}H NMR spectrum of aphidicol-16-ene (**8**) in $CDCl_3$

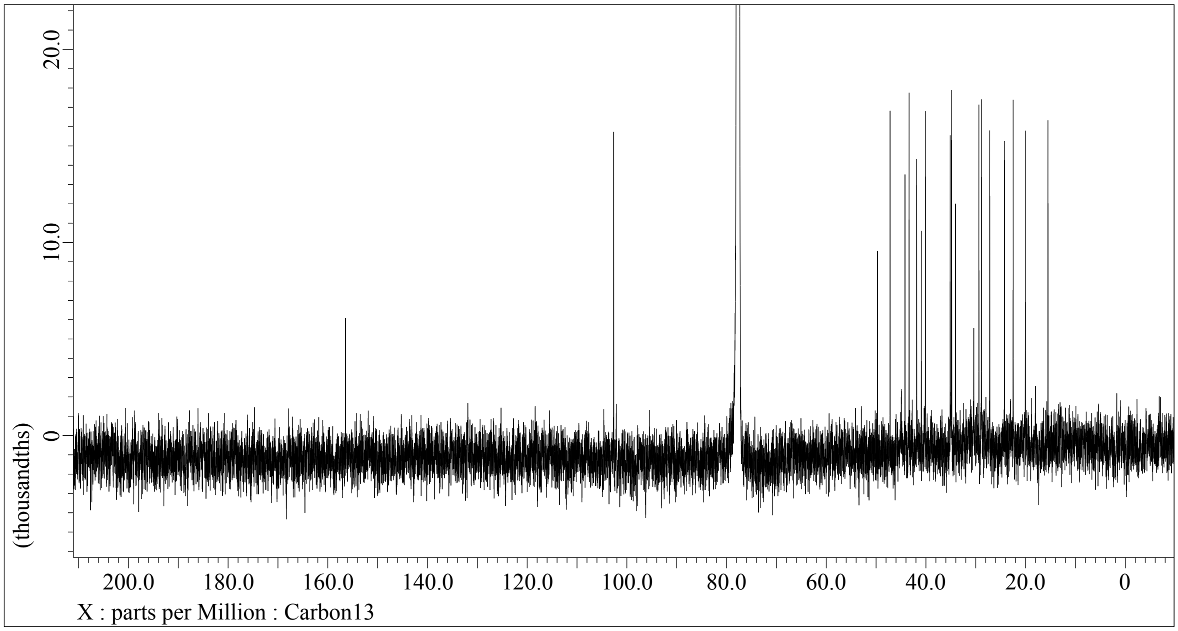

Figure S12: ^{13}C NMR spectrum of aphidicol-16-ene (**8**) in $CDCl_3$

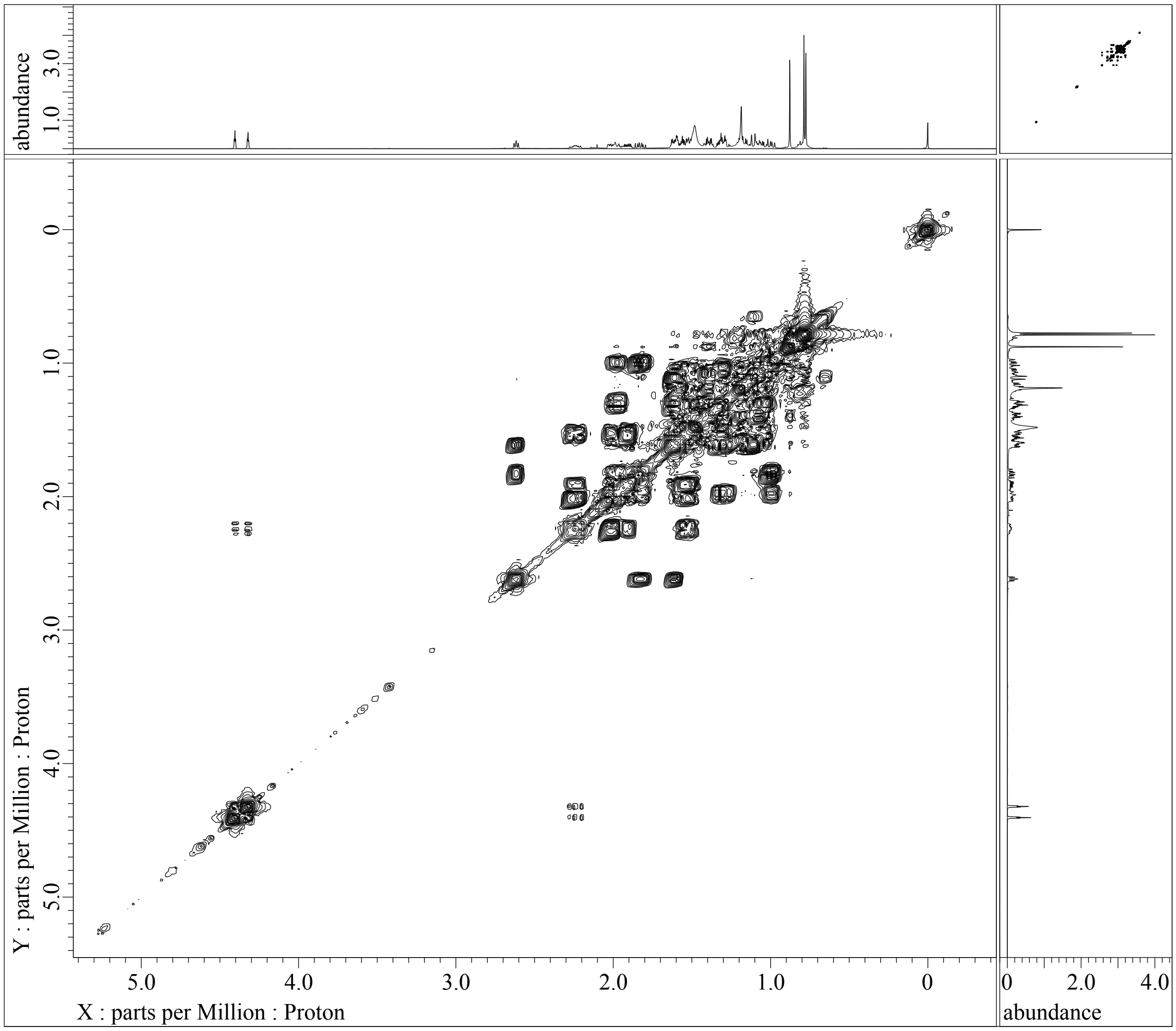

Figure S13: COSY spectrum of aphidicol-16-ene (**8**) in $CDCl_3$

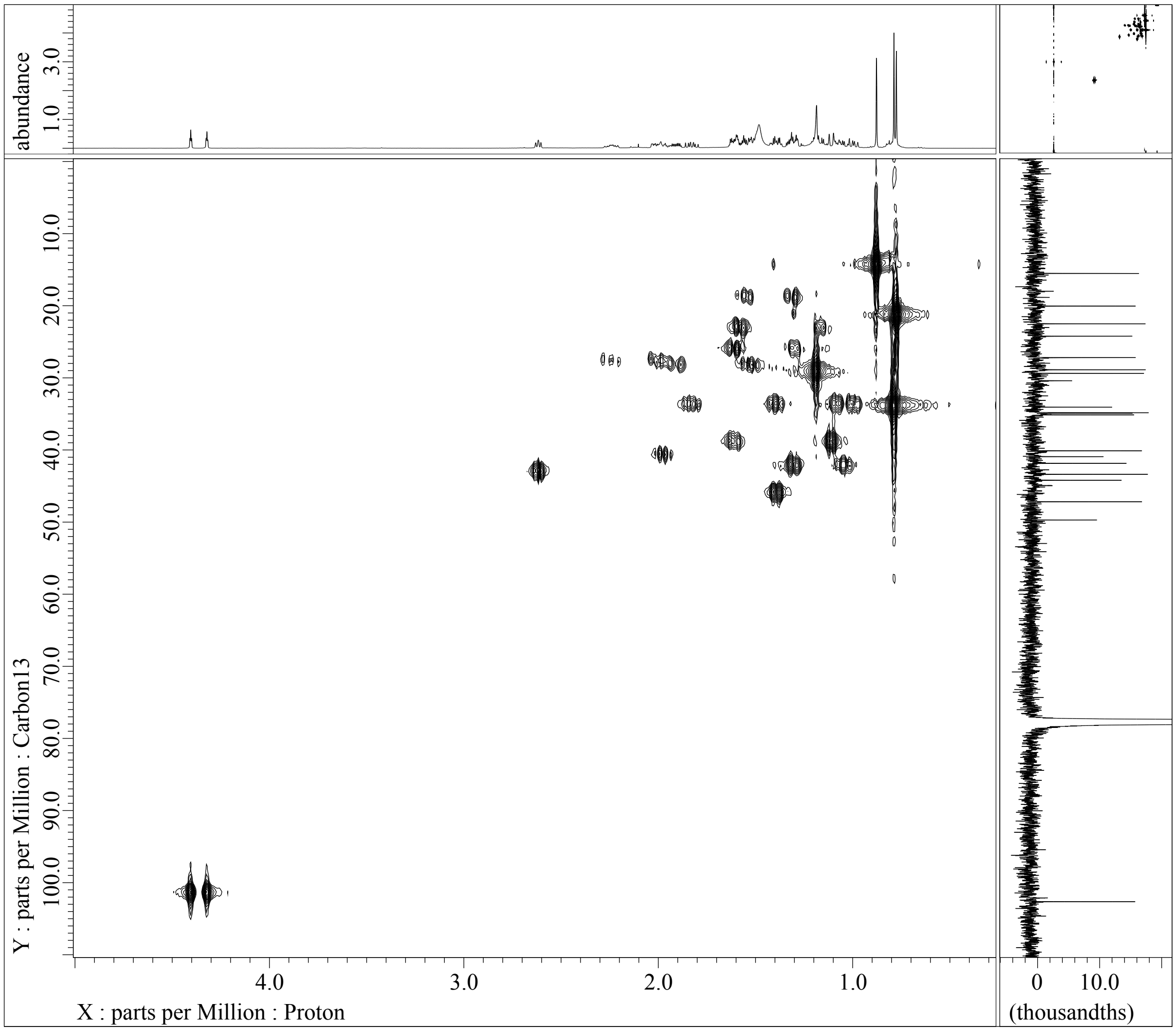

Figure S14: HMQC spectrum of aphidicol-16-ene (**8**) in $CDCl_3$

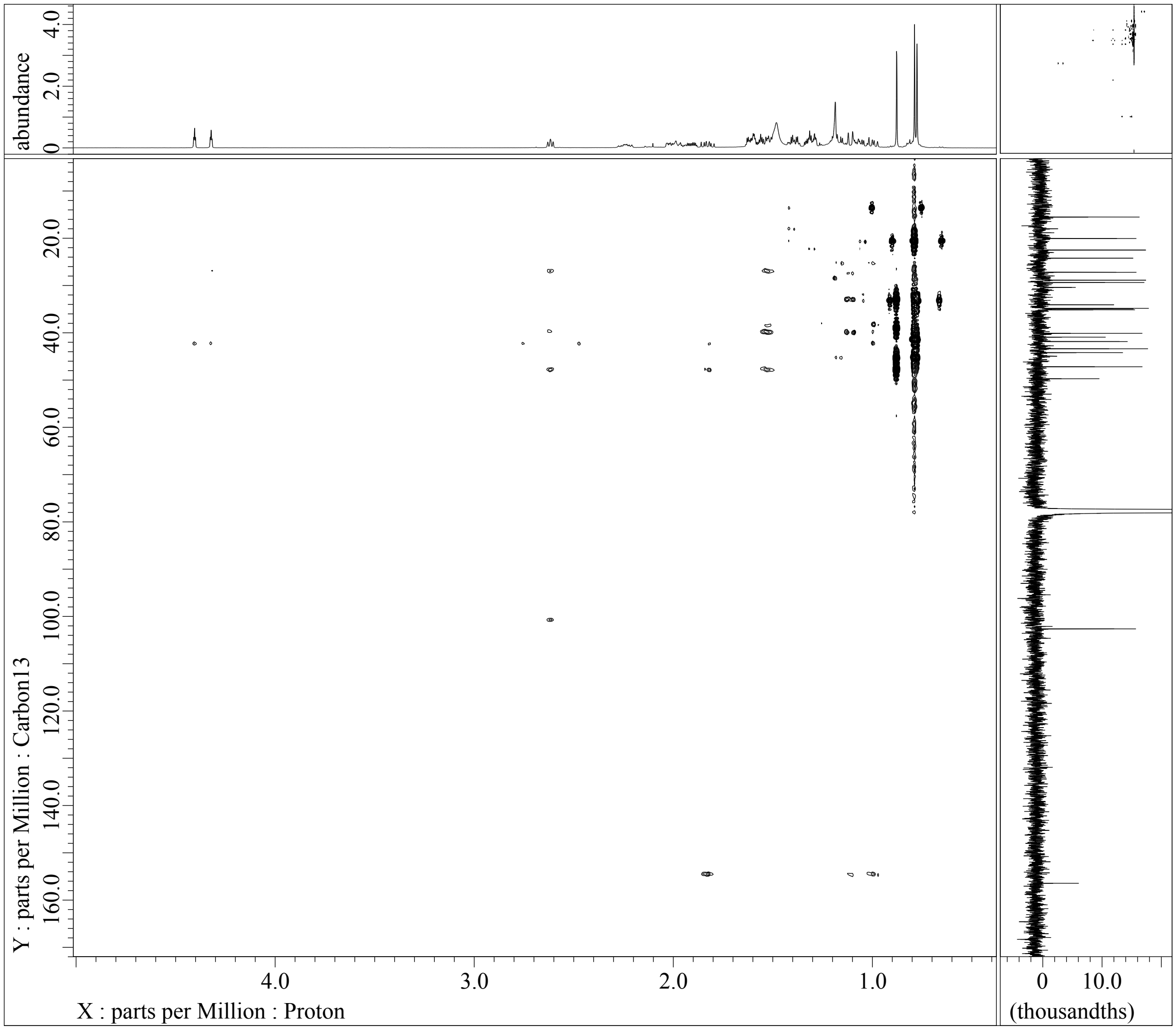

Figure S15: HMBC spectrum of aphidicol-16-ene (**8**) in $CDCl_3$

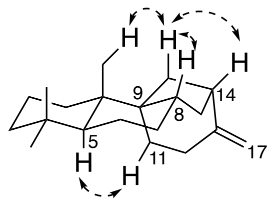

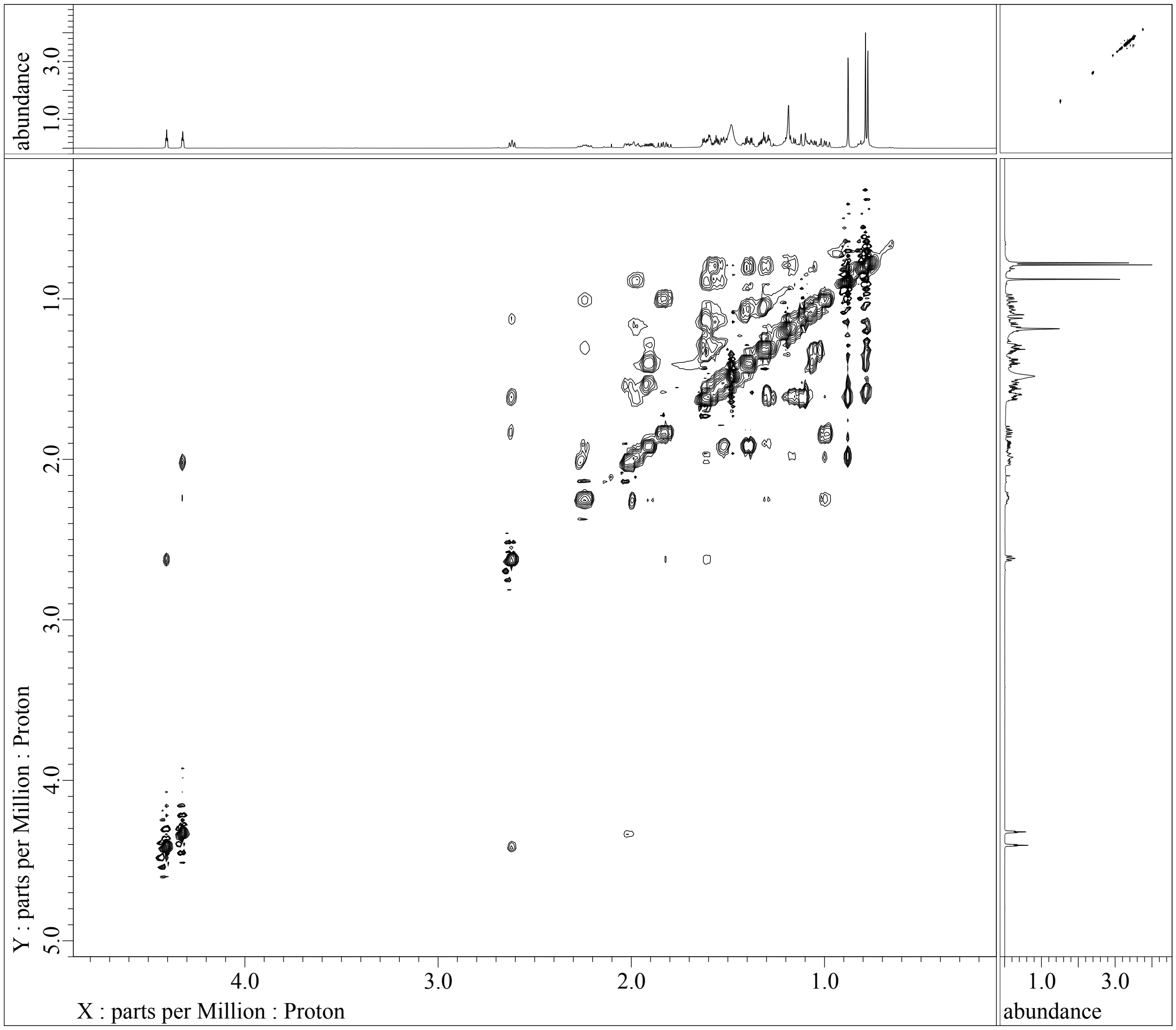

Figure S16: NOESY spectrum of aphidicol-16-ene (**8**) in $CDCl_3$

Additional Tables

Table S1. Primer sequences used in the present study

Primer name	Sequence
Cloning	
SdCPS2-Fw1	CTCAACATTTGGAGAGACACA
SdCPS2-Rv1	CCAGCAATGACTAGCTTGTTA
SdKSL1-Fw1	CTGGACCCGAGATACGTCAGTTTA
SdKSL1-Rv1	CGACATTGCAAGCAATTGTCTC
SdKSL2-Fw3	TACCAGACCGGAATCTGGA
SdKSL2-Rv5	TGGCCAACTTATGCGAAGAAA
Vector construction	
SdCPS2-NcoI-Fw	AGGAGATATACCATGGGCAGCTCGCACCTATCTGCA
SdCPS2-NotI-Rv	AAGCATTATGCGGCCGCTCACACGACTTTCTCAAAAAG
SdKSL1-NcoI-Fw	AGGAGATATACCATGGGCAGCATTCAAGATTCCACAGA
SdKSL1-NotI-Rv	ATGCTAGCTAATCTGGAAGGATTGGAA
SdKSL2-NcoI-Fw	AGGAGATATACCATGGGCAGCATTCAATATTCCACAGA
SdKSL2-NotI-Rv	TGCTCGAGTGCGGCCGCTCAGGTGGCCAACTTATGCGAAGA
SdCPS1-NcoI-Fw	AGGAGATATACCATGTCAAACAAGATATGGGAGAT
SdCPS1-NotI-Rv	AAGCATTATGCGGCCGCTTAGAGCACTCTTTCGAA
SmCPS-NcoI-Fw	AGGAGATATACCATGGCATGGATGGGCAGCAGT
SmCPS-NotI-Rv	GCATTATGCGGCCGCTCACGCGACTGGCTCGAA
Mutation	
SdKSL2-Stop232W-Fw	TGCAATTGGAATGAATTATTGAGCACT
SdKSL2-Stop232W-Rv	TTCATTCCAATTGCATGAATATTTTTC
qPCR	
UnivF-1131	AAACTTAAAGGAATTGACGG
18SrRNA-RV	GAAGGGATACCTCCGCATA
SdSCPS-126-FW	TATCTCATGCATGTGGCTGCTG
SdSCPS-224-RV	TCTGCCATATCCTGCAAAGCTG
SdKSL1-2282-FW	AAAGGAAGTGGGCTTTCACGAG
SdKSL1-2376-RV	TTTGTAAGCTCGTCCGACTGAC

Table S2. Abbreviations and accession numbers of DTSs

	Protein Name	Origin	Accession	Ref.
AtECPS	*ent*-copalyl diphosphate synthase	*Arabidopsis thaliana*	NP_192187	1
AtKS	*ent*-kaurene synthase	*A. thaliana*	NP_178064	2
CaCPS	copalyl diophosphate synthase	*Coffea arabica*	ACQ99373	–*
CcC8S	copalyl-8-ol diphosphate synthase	*Cistus cretic* subsp. *creticus*	E2IHE0	3
CmCPS1	copalyl diphosphate synthase	*Cucurbita maxima*	BAC76429	–
CsCPS	copalyl diphosphate synthase	*Croton sublyratus*	BAA95612	–
HaCPS1	copalyl diphosphate synthase	*Helianthus annuus*	CBL42915	4
HaKSL2	kaurene synthase	*H. annuus*	CBL42917	4
HaKSL3	kaurene synthase	*H. annuus*	CBL42916	4
HiCAS	*cis*-abienol synthase	*Handroanthus impetiginosus*	PIN17551	–
IeCPS1	*ent*-copalyl diphosphate synthase	*Isodon eriocalyx*	G3E4M6	5
IeCPS2	*ent*-copalyl diphosphate synthase	*I. eriocalyx*	G3E4M4	5
IrKSL6	isopimaradiene synthase	*I. rubuescens*	A0A1Z3GCD1	6
IrTPS2	nezukol synthase	*I. rubuescens*	ARO38140	7
LsECPS1	copalyl diphosphate synthase 1	*Lactuca sativa*	BAB12440	8
LsKS1	*ent*-kaurene synthase 1	*L. sativa*	XP_023729094	–
MvCPS1	peregrinol diphosphate synthase	*Marrubium vulgare*	A0A075FAK4	9
MvCPS2	*ent*-copalyl diphosphate synthase	*M. vulgare*	A0A075F9Z3	9
MvCPS3	(+)-copalyl diphosphate synthase	*M. vulgare*	A0A075FA51	9
MvKS	*ent*-kaurene synthase	*M. vulgare*	A0A075FBT3	9
MvELS	9,13-epolylabda-14-ene synthase	*M. vulgare*	A0A075FAK4	9
NtLPPS	copal-8-ol diphosphate hydratase	*Nicotiana tabacum*	G3CCC0	10
NtCAS	*cis*-abienol synthase	*N. tabacum*	NP_001312911	10
OsCPS1	*ent*-copalyl diphosphate synthase 1	*Oryza sativa*	XP_015624005	11
OsCPS2	*ent*-copalyl diphosphate synthase 2	*O. sativa*	XP_015625954	11
OsCPS4	*syn*-copalyl diphosphate synthase	*O. sativa*	NP_001389265	11
OsKS1	*ent*-kaur-16-ene synthase	*O. sativa*	Q0JA82	12
OsKSL4	9-b-pimara-7,15-diene synthase	*O. sativa*	NP_001389270	13
OsKSL5	*ent*-pimara-8,15-diene synthase	*O. sativa*	NP_001388808	13
OsKSL6	*ent*-isokaur-15-ene synthase	*O. sativa*	NP_001388811	13
OsKSL7	*ent*-cassa-12,15-diene synthase	*O. sativa*	XP_015622683	11
OsKSL8	stemar-13-ene synthase	*O. sativa*	XP_015617512	11
OsKSL10	*ent*-sandaracopimara-8,15-diene synthase	*O. sativa*	NP_001391661	13
PtKS	ent-kaur-16-ene synthase	*Populus trichocarpa*	XP_024463572	–
RoCPS1	copalyl diphosphate synthase	*Salvia rosmarinus*	AHL67261	14
RoKSL1	kaurene synthase-like 1	*S. rosmarinus*	AHL67262	14
SdCPS1	*ent*-copalyl diphosphate synthase	*Scoparia dulcis*	BAD91286	15
SdCPS2	*syn*-copalyl dophosphate synthase	*S. dulcis*	ALI57121	16
SdKS	*ent*-kaurene synthase	*S. dulcis*	AEF33360	17
SdKSL1	scopadula-13α-ol synthase	*S. dulcis*	BAX34756	16
SdKSL2	kaurene synthase like	*S. dulcis*	BBK15474	16

* unpublished.

Table S2. continued.

SmCPS1	copalyl diphosphate synthase	*Salvia miltiorrhiza* f. *alba*	AHJ59321	18
SmCPS2	copalyl diphosphate synthase 2	*S. miltiorrhiza* f. *alba*	AHJ59322	18
SmCPS3	copalyl diphosphate synthase 3	*S. miltiorrhiza* f. *alba*	AHJ59323	18
SmCPS5	*ent*-copalyl diphosphate synthase	*S. miltiorrhiza* f. *alba*	AHJ59324	18
SmKSL1	miltiradiene synthase	*S. miltiorrhiza* f. *alba*	C8XPS0	19
SmKSL2	kaurene synthase 2	*S. miltiorrhiza* f. *alba*	AHJ59325	18
SlCPS	copalyl diphosphate synthase	*Solanum lycopersicum*	BAA84918	–
SrCPS1	copalyl diphosphate synthase	*Stevia rebaudiana*	AAB87091	20
SrKS1	*ent*-kaurene synthase 1	*S. rebaudiana*	Q9XEH9	20
SsLPPS	copal-8-ol diphosphate hydratase	*Salvia sclarea*	G8GJ95	21
SsSS	sclareol synthase	*S. sclarea*	AFU61898	22
SsTPS3	diterpene synthase 3	*S. sclarea*	K4IAL8	21
VacTPS1	peregrinol diphosphate synthase	*Vitex agnus-castus*	A0A2K9RFZ7	23
VacTPS2	class I diterpene synthase 2	*V. agnus-castus*	A0A2K9RFZ2	23
VacTPS3	*syn*-copalyl diphosphate synthase	*V. agnus-castus*	A0A2K0RFZ8	23
VacTPS4	*ent*-kaurene synthase	*V. agnus-castus*	A0A2K9RFY0	23
VacTPS5	kolavenyl diphosphate synthase	*V. agnus-castus*	A0A2K0RG07	23
VacTPS6	class I diterpene synthase 6	*V. agnus-castus*	A0A2K9RFZ9	23
PpCPS/KS	*ent*-kaurene synthase	*Physcomitrium patens*	BAF61135	24

* unpublished

References

1. Mayer, K. *et al.*, Sequence and analysis of chromosome 4 of the plant *Arabidopsis thaliana. Nature* **1999**, *402*, 769–777. doi: 10.1038/47134
2. Theologis, A. *et al.*, Sequence and analysis of chromosome 1 of the plant *Arabidopsis thaliana*. *Nature* **2000**, *408*, 816–820. doi: 10.1038/35048500
3. Falare, V.; Pichersky, E.; Kanellis, A.K. A copal-8-ol diphosphate synthase from the angiosperm *cistus creticus* subsp. *creticus* is a putative key enzyme for the formation of pharmacologically active, oxygen-containing labdane-type diterpenes. *Plant Physiol* **2010**, *154*, 301–310. doi: 10.1104/pp.110.159566
4. Pugliesi, C.; Fambrini, M.; Salvini, M. Molecular cloning and expression profile analysis of three sunflower (*Helianthus annuus*) diterpene synthase genes. *Biochem Genet* **2011**, *49*, 46–62. doi: 10.1007/s10528-010-9384-6
5. Li, J.; Chen, Q.; Jin, Q.; Gao, J.; Zhao, P.; Lu, S.; Zeng, Y. IeCPS2 is potentially involved in the biosynthesis of pharmacologically active Isodon diterpenoids rather than gibberellin. *Phytochemistry* **2012**, *76*, 32–39. doi: 10.1016/j.phytochem.2011.12.021
6. Jin, B.; Cui, G.; Guo, J.; Tang, J.; Duan, L.; Lin, H.; Shen, Y.; Chen, T.; Zhang, H.; Huang, L. Functional diversification of kaurene synthase-like genes in *Isodon rubescens*. *Plant Physiol* **2017**, *174*, 943–955. doi: 10.1104/pp.17.00202
7. Pelot, K.A.; Hagelthorn, D.M.; Addison, J.B.; Zerbe, P. Biosynthesis of the oxygenated diterpene nezukol in the medicinal plant *Isodon rubescens* is catalyzed by a pair of diterpene synthases. *PLoS One* **2017**, *12*, e0176507. doi:10.1371/journal.pone.0176507
8. Sawada, Y., Katsumata, T.; Kitamura, J.; Kawaide, H.; Nakajima, M.; Asami, T.; Nakaminami, K.; Kurahashi, T.; Mitsuhashi, W.; Inoue, Y.; Toyomasu, T. Germination of photoblastic lettuce seeds is regulated via the control of endogenous physiologically active gibberellin content, rather than of gibberellin responsiveness. *J Exp Bot* **2008**, *59*, 3383–3393. doi: 10.1093/jxb/ern192
9. Zerbe, P.; Chiang, A.; Dullat, H; O'Neil-Johnson, M; Starks, C.; Hamberger, B.; Bohlmann, J. Diterpene synthases of the biosynthetic system of medicinally active diterpenoids in *Marrubium vulgare*. *Plant J* **2014**, *79*, 914–927. doi:10.1111/tpj.12589

10. Sallaud, C.; Giacalone, C.; Töpfer, R.; Goepfert, S.; Bakaher, N.; Rösti, S.; Tissier, A. Characterization of two genes for the biosynthesis of the labdane diterpene Z-abienol in tobacco (Nicotiana tabacum) glandular trichomes. *Plant J* **2012**, *72*, 1–17. doi: 10.1111/j.1365-313X.2012.05068.x
11. Kawahara Y. *et al.* Improvement of the *Oryza sativa* Nipponbare reference genome using next generation sequence and optical map data. *Rice* **2013**, *6*, 4. doi: 10.1186/1939-8433-6-4
12. Margis-Pinheiro, M.; Zhou, X.; Zhu, Q.; Dennis, E.S.; Upadhyaya, N.M. Isolation and characterization of a Ds-tagged rice (Oryza sativa L.) GA-responsive dwarf mutant defective in an early step of the gibberellin biosynthesis pathway. *Plant Cell Rep* **2005**, *23*, 819–833. doi: 10.1007/s00299-004-0896-6
13. Rice Full-Length cDNA Consortium. Collection, mapping, and annotation of over 28,000 cDNA clones from japonica rice. *Science* **2003**, *301*, 376–379. doi: 10.1126/science.1081288
14. Brückner, K.; Bozic, D.; Manzano, D.; Papaefthimiou, D.; Pateraki, I.; Scheler, U.; Ferrer, A.; de Vos, R.C.H.; Kanellis, A.K.; Tissier, A. Characterization of two genes for the biosynthesis of abietane-type diterpenes in rosemary (Rosmarinus officinalis) glandular trichomes. *Phytochemistry* **2014**, *101*, 52–64. doi: 10.1016/j.phytochem.2014.01.021
15. Nakagiri, T.; Lee, J.-B.; Hayashi, T. cDNA cloning, functional expression and characterization of *ent*-copalyl diphosphate synthase from *Scoparia dulcis* L. *Plant Sci* **2005**, *169*, 760–767. doi: 10.1016/j.plantsci.2005.05.028
16. Yamamura, Y.; Kurosaki, F.; Lee, J.-B. Elucidation of terpenoid metabolism in *Scoparia dulcis* by RNA-Seq analysis. *Sci Rep* **2017**, *7*, 43311, doi:10.1038/srep43311.
17. Yamamura, Y.; Taguchi, Y.; Ichitani, K.; Umebara, I; Ohshita, A.; Kurosaki, F.; Lee, J.-B. Characterization of *ent*-kaurene synthase and kaurene oxidase involved in gibberellin biosynthesis from *Scoparia dulcis. J Nat Prod* **2018,** *72*, 456–463. doi: 10.1007/s11418-017-1168-4
18. Cui, G.; Duan. L.; Jin, B.; Qian, J.; Xue, Z.; Shen, G.; Snyder, J.H.; Song, J.; Chen, S.; Huang, L.; Peters, R.J.; Qi, X. Functional divergence of diterpene synthases in the medicinal plant *Salvia miltiorrhiza*. *Plant Physiol* **2015**, *169*, 1607–1618. doi: 10.1104/pp.15.00695
19. Gao, W.; Hillwig, L.H.; Huang, L.; Cui, G.; Wang, X.; Kong, J.; Yang, B.; Peters, R.J. A functional genomics approach to tanshinone biosynthesis provides stereochemical insights. *Org Lett* **2009**, *11*, 5170–5173. doi: 10.1021/ol902051v.
20. Richman, A.S.; Gijzen, M.; Starratt, A.N.; Yang, Z.; Brandle, J.E. Diterpene synthesis in *Stevia rebaudiana*: recruitment and up-regulation of key enzymes from the gibberellin biosynthetic pathway. *Plant J* **1999**, *19*, 411-421. doi: 10.1046/j.1365-313x.1999.00531.x
21. Schalk, M.; Pastore, L.; Mirata, M.A.; Khim, S.; Schouwey, M.; Deguerry, F.; Pineda, V.; Rocci, L.; Daviet, L. Toward a biosynthetic route to sclareol and amber odorants. *J Am Chem Soc* **2012**, *134*, 18900–18903. doi: 10.1021/ja307404u
22. Caniard, A.; Zerbe, P.; Legrand, S.; Cohade, A.; Valot, N.; Magnoard, J.L.; Bohlmann, J.; Legendre. L. Discovery and functional characterization of two diterpene synthases for sclareol biosynthesis in *Salvia sclarea* (L.) and their relevance for perfume manufacture. *BMC Plant Biol* **2012**, *12,* 119. doi: 10.1186/1471-2229-12-119
23. Heskes, A.M.; Sundram, T.C.M.; Boughton, B.A.; Jensen, N.B.; Hansen, N.L.; Crocoll, C.; Cozzi, F.; Rasmussen, S.; Hamberger, B.; Hamberger, B.; Staerk, D.; Møller, B.L.; Pateraki, I. Biosynthesis of bioactive diterpenoids in the medicinal plant *Vitex agnus-castus*. *Plant J* **2018**, *93*, 943–958. doi: 10.1111/tpj.13822
24. Hayashi, K.; Kawaide, H.; Notomi, M.; Sakigi, Y.; Matsuo, A.; Nozaki, H. Identification and functional analysis of bifunctional *ent*-kaurene synthase from the moss *Physcomitrella patens. FEBS Lett* **2006** *580*, 6175–6181. doi: 10.1016/j.febslet.2006.10.018

Table S3: ^{1}H and ^{13}C NMR assignments of *syn*-copalol (**3'**) in $CDCl_3$

Position		δ_H	δ_C
1	a	1.57 (1H, m)	36.8
	b	1.05 (1H, m)	
2	a	1.61 (1H, m)	19.2
	b	1.45 (1H, m)	
3	a	1.38 (1H, m)	42.7
	b	1.17 (1H, m)	
4			33.2
5		1.26 (1H, m)	45.8
6	a	1.59 (1H, m)	23.7
	b	1.30 (1H, m)	
7	a	2.17 (1H, m)	31.6
	b	2.07 (1H, m)	
8			149.2
9		1.50 (1H, m)	57.9
10			38.0
11	a	1.62 (1H, m)	24.5
	b	1.47 (1H, m)	
12	a	1.90 (1H, m)	38.2
	b	1.74 (1H, m)	
13			140.6
14		5.41 (1H, m)	122.9
15		4.16 (2H, d, *J*=6.9 Hz)	59.5
16		1.67 (3H, br. s)	16.5
17	a	4.69 (1H, br. t, *J*=2.5 Hz)	109.5
	b	4.51 (1H, br. t, *J*=1.9 Hz)	
18		0.87 (3H, s)	33.5
19		0.81 (3H, s)	22.2
20		0.92 (3H, s)	22.4

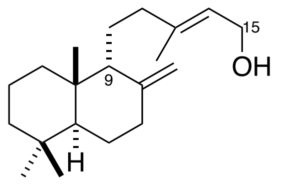

Table S4: ^{1}H and ^{13}C NMR assignments of scopadula-13α-ol (**4**) in $CDCl_3$

Position		δ_H	δ_C
1		1.39 (2H, m)	32.4
2	a	1.56 (1H, m)	18.5
	b	1.41 (1H, m)	
3	a	1.34 (1H, m)	42.0
	b	1.07 (1H, m)	
4			32.8
5		0.88 (1H, m)	47.8
6	a	1.45 (1H, m)	21.6
	b	1.30 (1H, m)	
7		1.48 (2H, m)	30.0
8		1.78 (1H, m)	36.9
9			52.3
10			38.3
11	a	1.44 (1H, m)	44.1
	b	0.91 (1H, m)	
12			43.9
13		3.35 (1H, t,5.2 Hz)	76.1
14	a	1.78 (1H, m)	38.0
	b	0.88 (1H, m)	
15	a	1.70 (1H, m)	24.2
	b	1.37 (1H, m)	
16	a	1.65 (1H, m)	30.5
	b	1.10 (1H, m)	
17		0.97 (3H, s)	23.1
18		0.78 (3H, s)	21.8
19		0.79 (3H, s)	33.4
20		0.94 (3H, s)	17.1

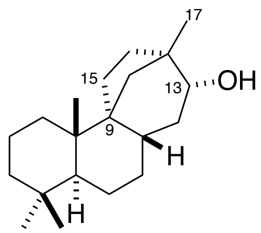

Table S5: ^{1}H and ^{13}C NMR assignments of aphidicol-16-ene (**8**) in $CDCl_3$

Position		δ_H	δ_C
1	a	1.42 (1H, m)	34.1
	b	1.13 (1H, m)	
2	a	1.59 (1H, m)	19.2
	b	1.36 (1H, m)	
3	a	1.35 (1H, m)	42.6
	b	1.09 (1H, m)	
4			33.4
5		1.44 (1H, m)	46.4
6	a	1.63 (1H, m)	23.5
	b	1.22 (1H, m)	
7	a	1.67 (1H, m)	26.4
		1.22 (1H, m)	
8		2.04 (1H, m)	40.9
9			49.0
10			40.2
11	a	1.94 (1H, m)	28.6
	b	1.58 (1H, m)	
12	a	2.28 (1H, m)	28.0
	b	2.09 (1H, m)	
13			155.8
14		2.67 (1H, t, 2.3)	43.5
15	a	1.89 (1H, m)	34.0
	b	1.02 (1H, m)	
16	a	1.66 (1H, m)	39.3
	b	1.16 (1H, m)	
17	a	4.46 (1H, t, 2.3)	101.7
	b	4.37 (1H, t, 2.3)	
18		0.84 (3H, s)	34.2
19		0.83 (3H, s)	21.5
20		0.93 (3H, s)	14.7

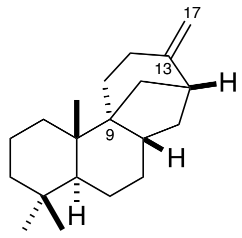

Supplement: Supplementary file 1 [file plants-12-00069-s001.zip › plants-2133603-supplementary.pdf]
